# All-optical adaptive control of quantum cascade random lasers

**DOI:** 10.1038/s41467-020-19305-8

**Published:** 2020-11-02

**Authors:** S. Schönhuber, N. Bachelard, B. Limbacher, M. A. Kainz, A. M. Andrews, H. Detz, G. Strasser, J. Darmo, S. Rotter, K. Unterrainer

**Affiliations:** 1 Photonics Institute, TU Wien, 1040 Vienna, Austria; 2 Center for Micro- and Nanostructures, TU Wien, 1040 Vienna, Austria; 3 Institute for Theoretical Physics, TU Wien, 1040 Vienna, Austria; 4 Institute for Solid-State Electronics, TU Wien, 1040 Vienna, Austria; 5 Central European Institute of Technology, Brno University of Technology, 61200 Brno, Czech Republic

**Keywords:** Quantum cascade lasers, Terahertz optics, Photonic devices

## Abstract

Spectral fingerprints of molecules are mostly accessible in the terahertz (THz) and mid-infrared ranges, such that efficient molecular-detection technologies rely on broadband coherent light sources at such frequencies. If THz Quantum Cascade Lasers can achieve octave-spanning bandwidth, their tunability and wavelength selectivity are often constrained by the geometry of their cavity. Here we introduce an adaptive control scheme for the generation of THz light in Quantum Cascade Random Lasers, whose emission spectra are reshaped by applying an optical field that restructures the permittivity of the active medium. Using a spatial light modulator combined with an optimization procedure, a beam in the near infrared (NIR) is spatially patterned to transform an initially multi-mode THz random laser into a tunable single-mode source. Moreover, we show that local NIR illumination can be used to spatially sense complex near-field interactions amongst modes. Our approach provides access to new degrees of freedom that can be harnessed to create broadly-tunable sources with interesting potential for applications like self-referenced spectroscopy.

## Abstract (web summary)

Tunable quantum cascade lasers can enable applications in multiple areas. Here, the authors demonstrate the adaptive control of the modes and emission spectra of quantum cascade random lasers through a spatially-tailored optical modulation of the active region.

## Introduction

The sensing of well-defined molecular transitions is crucial in various domains such as gas spectroscopy. Typically, transitions correspond to narrow absorption lines, which, in order to be probed, rely on carefully designed THz quantum cascade laser (QCL) sources that are manufactured through high-precision processes. Sweeping the frequency across the absorption spectrum of molecules requires, moreover, a tuning mechanism both to adjust the wavelength and to correct built-in inaccuracies. Various technological solutions have been proposed to tune QCLs. Among them, slight frequency tuning was demonstrated using electrically driven heat sinks to modify the refractive index of the active region^1^. External optical cavities coupled to QCLs have been employed to realize continuous tuning ranges from 12 to 50 GHz^2–5^, while more substantial ranges of 67 and 240 GHz were achieved using micro-optomechanics^6,7^. Remarkably, an optical tuning mechanism relying on near-infrared (NIR) laser excitation was recently exploited by several groups to manipulate the output power and frequency of mid-infrared QCLs. For QCLs ridge resonators under NIR illumination, tuning ranges of several GHz were reported^8,9^ and put to use for remarkable applications in molecular spectroscopy^8^.

**Fig. 1:**
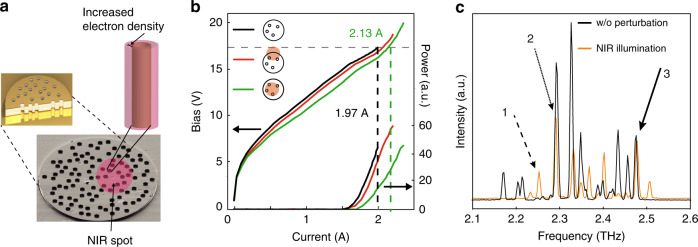
QCRLs under NIR-spot scanning. **a** SEM picture of a QCRL with a NIR laser spot scanned across its surface (wavelength 813 nm, diameter 270 µm, red circle). The QCRL (diameter 1 mm) is filled with holes (diameter 20 µm) for a filling fraction of 18%. A schematic cross-section of the device is provided in the inset. The NIR light is absorbed and creates electron-hole pairs along the edges of the holes (right-hand-side schematic). The generated pairs produce a local increase in carrier density (red cylinder around the hole), which exponentially decays away from the hole. **b** Light–current–voltage characteristics of the QCRL without perturbation (black), for a spot in the center (green) and at the edge (red) of the sample (insets). Under a voltage bias of 17.5 V (horizontal gray dashed line), the current increases from 1.97 A (vertical black dashed line) to a maximum of 2.13 A (vertical green dashed line) due to photoexcited electrons. **c** Spectra measured in the absence of the perturbation (“w/o perturbation”, black curve) and in the presence of the NIR perturbation spot (“NIR illumination”, orange curve) at position (*x*, *y*) = (6, 9). The mode structure is influenced by the NIR illumination. New modes emerge at seemingly arbitrary frequencies (“1”, dashed line arrow), while existing modes are either attenuated (“2”, dotted line arrow) or unaffected (“3”, solid line arrow).

Recently, quantum cascade random lasers (QCRLs) that intrinsically feature multi-mode emission^10–15^ were demonstrated (Fig. 1a). Random lasers define a class of lasers whose feedback mechanism relies on a disordered gain medium as opposed to conventional cavities^16–18^. The far field of these devices can be collimated^11^, which is particularly remarkable since QCLs are typically characterized by a large output divergence through diffraction that is caused by the mismatch between cavity dimensions and wavelength. The multiple-scattering events in these QCRLs are induced by randomly placed air holes (etched through the active region), resulting in lasing spectra without equally spaced modes. Thus, modifying the scattering inside the active region allows one to tune the THz emission spectrum of QCRLs. Moreover, since gain competition and spatial hole burning are actively present in the selection or suppression of lasing modes in QCLs, QCRLs represent a promising playground to collect information about nonlinear interactions among modes.

Here, we demonstrate a control scheme in the context of QCRLs, in which the permittivity is spatially modulated through an intensity-shaped optical beam. An external NIR laser beam is coupled into the structure through the randomly placed air holes in the QCRL and thereby changes the permittivity in the vicinity of the holes. Using a spatial light modulator, the distribution of NIR perturbation is iteratively optimized to reshape the emission of the device, which, being initially multi-mode, is transformed into a tunable single-mode source. Our approach points the way towards addressing major challenges in QCLs, such as near-field probing or the investigation of nonlinear modal interactions (e.g., spatial hole burning). Harnessing the many degrees of freedom available in the spatial pattern of the NIR beam, our approach demonstrates an all-optical controllability in QCRLs, which enables the reshaping of THz light to realize unconventional spectral features like bi-color emission. We envision that this technique can be straightforwardly extended to tune also other properties like lasing directivity.

## Results

### Optical tuning of QCRLs through local NIR perturbation

Our sample consists of an electrically driven QCRL manufactured by etching holes into an active region composed of superimposed GaAs/Al_0.15_Ga_0.85_As layers (Fig. 1a and “Methods”). The holes of diameter 20 µm are randomly distributed with a filling fraction of 18% and provide both the confinement of the modes (through a spatially disordered permittivity), as well as the outcoupling of radiation around 2.3 THz. The lasing modes are typically extended over the device (Supplementary Fig. 1) while the THz field they emit is collected through a Fourier-transform infrared spectrometer (FTIR) coupled via parabolic mirrors. The chip is illuminated by a near-infrared (NIR) tunable Ti:Sapphire femtosecond laser. In a first set of measurements, we focus the beam on a perturbation spot (270 µm in diameter, red circle in Fig. 1a) that is spatially scanned across the surface of the device (1 mm in diameter) with an *x*−*y* translation stage (Supplementary Fig. 2a).

The perturbation spot induces a strong tuning of local permittivity within the QCRL. Regardless of its position on the device, when the frequency of the NIR beam is brought above the bandgap of GaAs (Ti:Sapphire’s set to 813 nm), we observe in the QCRL an additional photocurrent characteristic of the creation of electron-hole pairs (Supplementary Fig. 3). This feature emphasizes that—through changes in carrier concentration and conductivity—the spot locally excites an electron-hole plasma, which modifies the permittivity of the semiconductor. With a 1 µm penetration depth into the semiconductor, this NIR-induced refractive-index tuning is usually weak in QCLs^13^. In our QCRL, however, the spot illuminates a large area around the etched holes (Fig. 1a) and thereby produces substantial changes in the local permittivity, whose amplitude can be estimated from current variations. Under NIR illumination, the photoexcited carriers increase the current at a constant bias of 17.5 V (horizontal gray dashed line in Fig. 1b) from 1.97 A to 2.13 A (vertical black and green dashed lines in Fig. 1b, respectively). From the laser doping, the initial electron density is estimated close to *n*_*e*_ = 3.8 × 10^15^ cm^−3^. At the exposed areas around the holes, the density increases up to *n*_*e*_ = 2.4 × 10^16^ cm^−3^. This change in electron density shifts the plasma frequency of GaAs close to the emission frequency of the QCRL and small modifications translate into dramatic changes in permittivity according to Drude’s model (Methods). Initially at a value of *ϵ* = 12.9, under NIR illumination the permittivity decreases locally down to *ϵ* = 8.0. Thermal effects induced by NIR-beam heating are discussed in Supplementary Note 4 and turn out to be negligible (Supplementary Fig. 4).

While the local tuning of permittivity affects the amplitudes of the lasing modes and may turn them on and off, the spectral positions of lasing frequencies remain, however, largely preserved. This robustness of lasing frequencies against varying conditions can already be observed when increasing the QCL bias voltage without the perturbation by the NIR beam (Supplementary Fig. 5). Increasing the available gain in this way changes the amplification of pre-existing modes, while other modes are brought above threshold and start to lase—but no strong spectral shifts of modes are observed. Similarly, scanning the NIR beam across the QCRL under constant bias also preserves the lasing frequencies across the entire 350 GHz-span spectrum (Supplementary Fig. 6). Fig. 1c displays the spectra of a non-perturbed (“w/o perturbation”, black curve) and an example of a perturbed QCRL (“NIR illumination”, orange curve) illuminated at location (*x*, *y*) = (6, 9) (Supplementary Fig. 2a and explanation therein). Although the spot illuminates only about 8 % of the surface, we observe in Fig. 1c the emergence (“1”, dashed line arrow) and the attenuation or amplification (“2”, dotted line arrow) of modes, while other modes vanish simultaneously. For specific locations of the spot, the modes whose spatial distributions do not overlap with the perturbation remain unaffected (“3”, solid line arrow). Apart from giving rise to small frequency shifts of the lasing peaks, through local modifications of the dielectric constant^1^ the NIR beam mainly induces a redistribution of the available gain among pre-existing modes. These modes, in turn, compete through complex interactions (as further explained in subsection “Probing the mode structure”) and can sometimes be brought below or above threshold, but their frequencies are largely pre-determined by the confinement in the active region and the randomly positioned holes in it. Another interesting observation concerns the NIR-induced changes of the electric field in the active region, which affects the alignment of energy levels and typically leads to a decrease of the mean spectral intensity (Supplementary Fig. 7).

### Mode selection

**Fig. 2:**
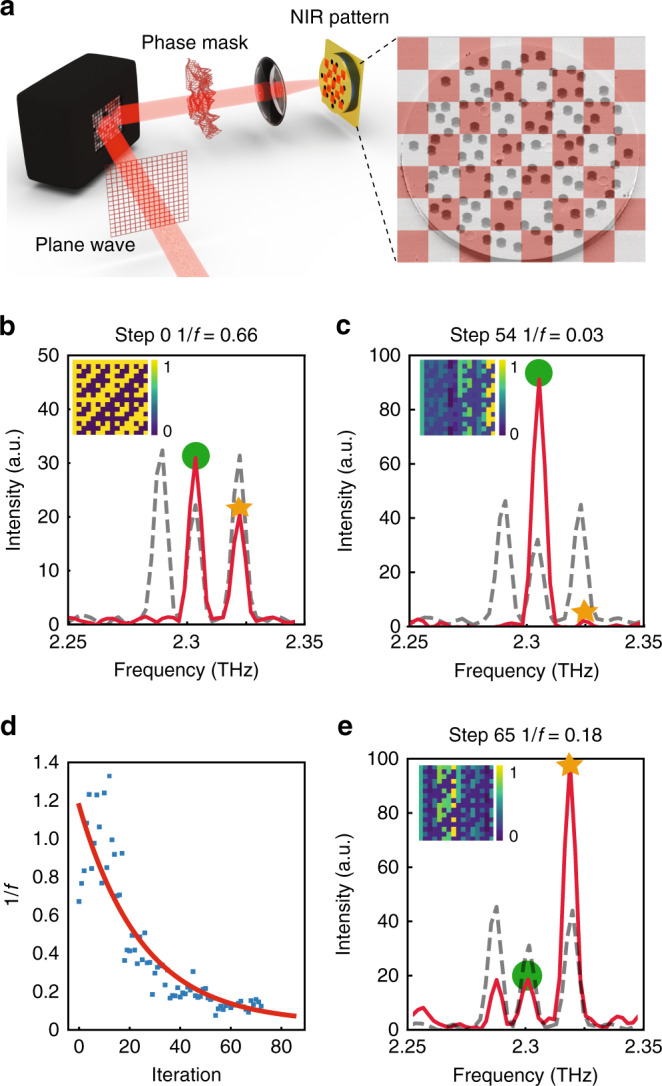
Mode selection in QCRLs through patterned NIR illumination. **a** Setup performing a patterned NIR illumination on the QCRL. The field emitted from the Ti:Sapphire (“Plane wave”) is phase-modulated by a spatial light modulator used in reflection mode (black box). The resulting field (“Phase mask”) is Fourier-transformed by a lens to produce an intensity pattern on the device (“NIR pattern”) (“Methods”). For illustration purposes, an example of a chess-board-like patterned illumination is provided in inset (right-hand side). **b** Starting point for the iteration of the optimization routine applied to a mode at 2.3 THz (Step 0). An initial non-uniform pattern is projected onto the QCRL (blue-yellow array in inset). The solid red curve corresponds to the spectrum collected under this non-uniform NIR illumination. The dashed gray curve shown here and in panels **c** and **e** was recorded without NIR perturbation. The green dot indicates the mode at 2.3 THz that the procedure intends to optimize, while the orange star indicates a second mode (at 2.32 THz) whose intensity should be reduced. **c** Final iteration of the optimization applied to the mode at 2.3 THz (Step 54). The algorithm converges towards a complex spatial modulation of the perturbation (blue-yellow array in inset) producing a single-mode emission at 2.3 THz (green dots). **d** Evolution of the function 1/*f* for the optimization displayed in **b** and **c**. **e** Final iteration of the optimization applied to a mode at 2.32 THz (step 65). The mode marked by an orange star (2.32 THz) is optimized by a non-uniform pattern (blue-yellow array in inset) obtained after 65 iterations.

The local tuning of permittivity provides access to new degrees of freedom, which can be put to use through an adaptive spatial patterning of the NIR illumination to reshape the disorder landscape and exercise precise control over the properties of the QCRL. For this purpose, the NIR beam is now spread over the whole device area (Supplementary Fig. 2b). Its intensity is then spatially modulated through a computer-controlled spatial light modulator (Fig. 2a) and an adapted non-uniform distribution modifies the spectrum of the QCRL. Inspired by former works^19–22^, we implement an iterative procedure to generate the NIR-perturbation spatial pattern (Methods), which selectively transforms the THz laser from multi-mode to single-mode through an adjusted depletion of specific modes. Yet, instead of relying on a modulation of optical gain, here the NIR beam is spatially modulated to restructure the permittivity of the system and, thus, its scattering disorder. Specifically, the QCRL is first electrically pumped until exhibiting three modes (dashed curves in Fig. 2b, c, and e). We then use a gradient-based optimization algorithm to reshape the NIR pattern through a nonlinear feedback loop, which aims to enhance the cost function $f = \frac{{I_{{\mathrm{opt}}}}}{{I_{{\mathrm{ref}}}}}$. Here, *I*_opt_ stands for the intensity of the desired mode and *I*_ref_ for the highest intensity among the remaining modes. Figure 2b displays the spectrum recorded during the first step (Step 0) of the optimization procedure dedicated to the selection of a mode located at 2.3 THz (green circle), together with the corresponding spatial NIR distribution in inset. Figure 2c shows the NIR pattern and the spectrum obtained after 54 iterations (Step 54), at which point we observe an almost-single-mode emission at frequency 2.3 THz with a cost function (identical to a rejection rate) close to 1/*f* = 16 dB. Figure 2d displays the evolution of 1/*f* throughout the optimization. In contrast with former works^19^, here the use of a gradient-based optimization offers a fast and efficient convergence (Supplementary Fig. 8 and “Methods”). The procedure can select any mode individually and, thus, provides tunability across the whole gain bandwidth at moderate bias current (“Methods”). In the present setup, the procedure operates based on the discrete distribution of initial modes; a continuous tuning would require, instead, a more weakly scattering sample or additional control parameters^8^. This point is illustrated in Fig. 2e, in which the QCRL used in Fig. 2b–d is optimized to emit at 2.32 THz (orange star) with 1/*f* = 7.5 dB.

This approach for the control of THz sources enables to create remarkable lasing features and can find straightforward applications in domains like molecular spectroscopy, in which lasing frequencies have to match the absorption spectra of specific elements. In particular, the QCRL emission range lies within the water-vapor absorption window (i.e., between 2.26 THz and 2.34 THz^21^) such that it can be used for the detection of molecules like HCN or NO_2_, which have dominant absorption lines at 2.319 THz and 2.298 THz, respectively^18^. As we show in the Supplementary Note 9, we reshape the spectrum within a given range to select and balance the intensities of two distinct modes, resulting in bi-modal emission (Supplementary Fig. 9). Our platform is thus naturally suited to perform self-referenced spectroscopic measurements, which require two lasing modes (one frequency being used to characterize the absorption of a molecule while another acts as a reference).

### Probing the mode structure

**Fig. 3:**
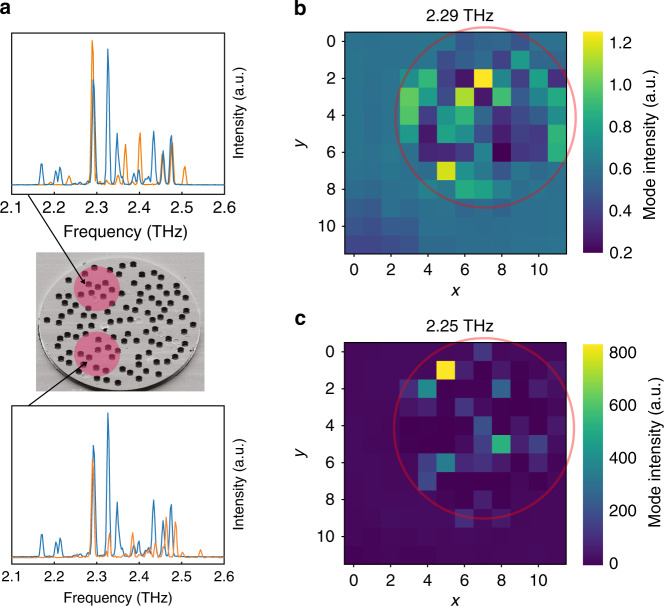
Mode mapping through spot scanning. **a** The NIR spot is scanned across the device and the resulting spectra are measured. The two panels on the right-hand side display examples of spectra (orange curves) collected for the two indicated spot positions (the blue curves are measured without perturbation). **b**, **c** Mode intensity (measured as a ratio with the unperturbed case) of individual modes at 2.29 and 2.25 THz, respectively, while the spot is scanned across the QCRL. Each pixel in the displayed 12 × 12 matrices is associated with a spot location (*x*, *y*) (the red circles mark the QCRLs’ outer rim).

One of the long-standing goals in the QCL community is to get experimental access to the spatial structure of lasing modes^23^—a task that has been hindered by the fact that in QCLs the near field is typically not accessible while the far-field emission contains only partial information regarding the lasing modes. Here, we demonstrate that local perturbations represent a powerful tool to collect data on modal extents over the device. Specifically, when scanning the NIR spot introduced in Fig. 1a across the QCRL (Supplementary Fig. 2a), intensity variations among modes follow drastically different patterns. For each position of the spot (Fig. 3a), we track the lasing modes by monitoring their frequencies. The evolutions of individual modes are then mapped into matrices—in which each one of the 12 × 12 elements corresponds to the intensity obtained for the NIR beam being located at a given location (*x*, *y*) among the 12 × 12 spot positions on the chip (“Methods”). Figure 3b, c displays the evolution while scanning the perturbation of a mode at 2.29 THz (label “2” in Fig. 1c) and at 2.25 THz (label “1” in Fig. 1c), respectively. The mode addressed in Fig. 3b emits for any spot position, but at different locations of the spot its intensity is strongly modulated while other modes are strongly impacted as well (Supplementary Fig. 10). In sharp contrast, the mode addressed in Fig. 3c does not emit in the spectrum collected without perturbation. At very specific spot positions (e.g., yellow spot at (*x*, *y*) = (5, 1) or green spot at (8, 5)) the mode starts lasing and the emissions of the remaining modes are strongly modified (Supplementary Fig. 11). In this way the mappings of Fig. 3b, c contain spatial information about the modes: each spot position defines a new gain distribution, which either amplifies or reduces the modes according to their spatial extent. From the complementarity of these two mappings, it can be concluded that the two modes probed in Fig. 3b, c describe rather different spatial so-called spheres of action, which characterize the scanning areas over which modes are strongly influenced by the NIR light and interact with each other.

**Fig. 4:**
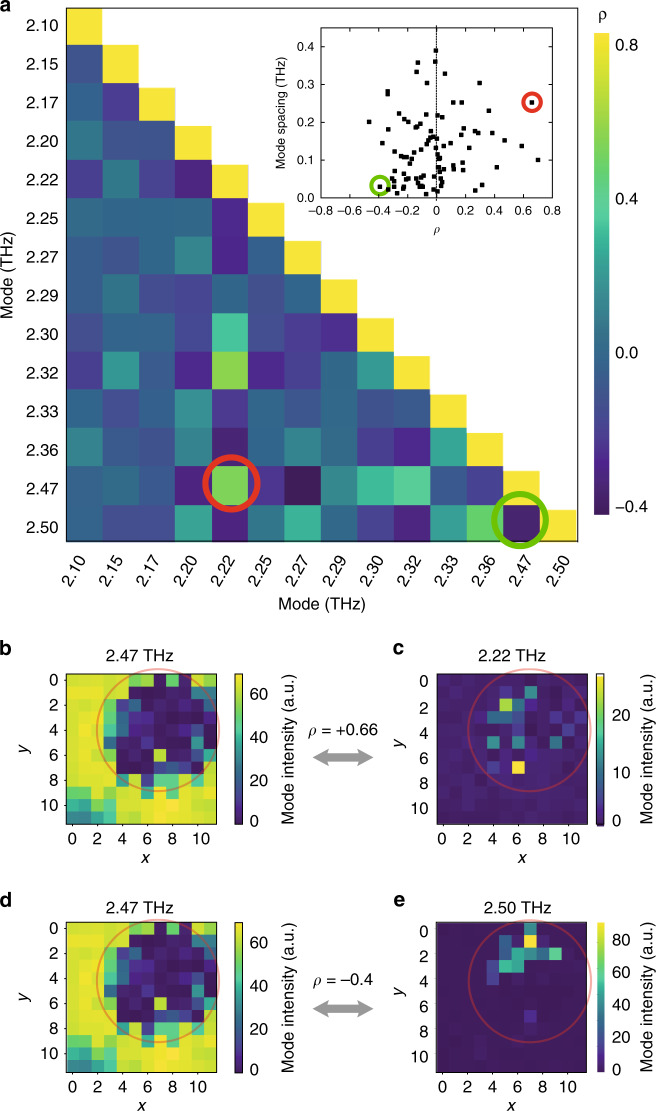
Correlation between mode mappings. **a** Pearson cross-correlation, *ρ*, between the mappings of 14 different modes. The red circle indicates a positive correlation of *ρ* = 66% for the modes at 2.47 and 2.22 THz, while the green circle pinpoints a negative correlation of *ρ* = −40% for the modes at 2.47 THz and 2.50 THz. For each pair of modes, the scattered plot in inset reports the cross-correlation between modes as a function of their frequency spacing. We observe a concentration of negative correlations for pairs close in frequency. The green and red circles correspond to the ones displayed in the main panel. **b**, **c** Mappings of the two modes at 2.47 and 2.22 THz, respectively, whose correlation is red-circled in **a**. **d**, **e** Mapping of the two modes at 2.47 THz and 2.50 THz, respectively, whose correlation is green-circled in **a**.

To quantify whether the spheres of action of two modes are similar or complementary, we study the mode mappings (like the one displayed in Fig. 3b, c) in terms of their cross-correlation. Figure 4a presents the Pearson cross-correlation, *ρ*, between 14 modes (“Methods”), in which positive values indicate similar evolutions under NIR illumination of the two selected modes (simultaneous amplification or attenuation), while negative values characterize inverted behaviors (simultaneous amplification of one mode and attenuation of the other). The inset panel displays the different correlations for the pairs of modes measured in Fig. 4a as a function of their frequency spacings. It emphasizes that modes close in frequency are typically spatially distinct and, thus, anti-correlate. We attribute this feature to the fact that modes that are not only spectrally, but also spatially very similar, tend to merge and thus appear as a single mode rather than as two distinct modes in the emission spectrum. Figure 4b, c depict two modes—at 2.47 and 2.22 THz, respectively—associated with a high-positive correlation value of *ρ* = 0.66 (red circle in Fig. 4a). Both modes display a similar response to NIR perturbations, which indicates that their modal intensity profiles do have a reinforcing spatial overlap with each other. In contrast, Fig. 4d, e display two modes—at 2.47 THz and 2.50 THz, respectively—associated with a negative correlation value of *ρ* = −0.4 (green circle in Fig. 4a). Here, the overlap between the modal intensity profiles is destructive as induced by strong gain competition.

The mappings shown in Figs. 3 and 4 provide an interesting glimpse into the modal structure of QCRLs, but they should not be mistaken as direct images of the modes. Instead, the intensities of individual modes are related to their spatial overlap with the gain and the intensity increases (respectively decreases) observed for specific scanning locations indicate a certain overlap (respectively mismatch) between the modes and the NIR beam. Yet, in QCLs, modes interact through different mechanisms and thus intensity variations can have multiple origins. More specifically, modal interactions can be caused by gain saturation, like spectral and spatial hole burning that have been reported to play a major role in QCLs’ mode selection^24–28^. QCLs also display strong nonlinear modal coupling that impacts their spectral organization^29,30^ or complex gain mechanisms such as inhomogeneous broadening or photon-assisted current^31^. Therefore, drawing a definitive conclusion on the modal extents as well as the origin of intensity variations will require further investigations (e.g., by applying our approach on simpler structures that are amenable to a numerical analysis).

## Discussion

We introduce an all-optical control mechanism for THz sources, which exploits the local excitation of electron-hole pairs to reshape the permittivity of the active region. Through an algorithmic scheme, an adaptively patterned NIR beam illuminating the device enables to select individual modes across a broad frequency range as well as to realize unconventional lasing features such as bi-color spectra. The spatial modulation of the permittivity provides access to yet unexplored degrees of freedom, which could be harnessed to control also other lasing properties like the directivity for multi-mode sources^21^ or to collect near-field information in THz lasers. Moreover, through the realization of unconventional spectral features our approach could offer new opportunities in areas like self-referenced spectroscopy and molecular detection with the advantage of being intrinsically compatible with various QCL technologies.

## Methods

### Fabrication

Quantum-cascade active regions are heterostructures formed by thin and alternating layers of different semiconductor materials. The created quantum wells form discrete energy levels that set emission frequencies. Here, the active region is realized with layers of GaAs and Al_0.15_Ga_0.85_As, which are grown by molecular beam epitaxy. The layers sequence of one period starting from the injector barrier is 4.3/8.9/2.46/8.15/4.17/1/5/10 nm, with a homogeneous n-doping with a density of 6·10^16^ cm^−3^ of the 5-nm well. The layer sequence is repeated 340 times, which leads to a thickness of the active region of 13 µm. A 100 nm highly doped GaAs layer at the bottom of the active improves the electrical contact. The active region and carrier substrate were covered with a 1 μm-thick gold layer, followed by an Au-Au thermo-compression bonding step. After lapping and wet etching, the gold-top contact layer was structured by a photo-lithography/lift-off process, acting as a self-aligned etch mask for the subsequent reactive ion etching process used to form the holes. The design of the holes (20 μm in diameter and 18% filling fraction) is selected to support spatially extended modes around 2.3 THz (i.e., wavelength of 130 µm). The device is then mounted on a copper heat sink with indium and contacted using a wire-bonding technique.

### Light–current–voltage (*L*–*I–V*) and spectrum measurements

Spectra of the sample are recorded using a Bruker Vertex 80 FTIR spectrometer with a resolution of 2.25 GHz, using an pyroelectric deuterated triglycine sulfate (DTGS) detector. The device is mounted in a liquid-helium-cooled-flow cryostat that is optically coupled to the spectrometer via parabolic mirrors. For *L*–*I*–*V* measurements, the light intensity *L* is acquired by feeding the FTIR-detector output (internal DTGS detector) into a lock-in amplifier (Stanford Research Systems SR830) and measuring the signal at the modulation frequency of 10 Hz. The current flowing through the device is measured with a coaxial current probe (Tektronix CT-1), which is connected to the output of a voltage pulser (HP8114A). The measurement data of the current *I* and voltage *V* are acquired using a digital oscilloscope (Tektronix DPO 3032).

### Near infrared illumination and light patterning

NIR illumination is provided by a Ti:Sapphire Mira 9000 laser system with both tunable wavelength and tunable output power, which operates in continuous mode. For an efficient perturbation of the QCRL, in the main text the wavelength is set to 813 nm with an intensity of 250 mW. In Figs. 1, 3, and 4, the laser is focused on a 270 µm -diameter spot and scanned across the device (Supplementary Fig. 2a). In Fig. 2, a phase-only spatial light modulator is used in reflection mode to modulate the Ti:Sapphire field and to form a phase mask (Supplementary Fig. 2b). With the spatial light modulator being located in the Fourier plane of the optical setup, the phase mask is Fourier-transformed by a lens to produce an intensity-modulated pattern on the device. The phase mask required to produce a specific intensity pattern is calculated using the Gerchberg-Saxton algorithm^28^.

The NIR illumination of a semiconductor heterostructure leads to the excitation of electron-hole pairs. The NIR light is coupled into the structure through the holes in the top gold layer. Here, we assume a simple rectangular excitation profile with a thickness of 1 µm (corresponding to the absorption length), which allows the estimation of the local electron density. The change in permittivity is calculated by applying Drude’s mode^32^

1 $${\it{\epsilon }}_{{\mathrm{THz}}} = {\it{\epsilon }}\left( {1 - \frac{{\omega _P^2}}{{\omega ^2}}} \right).$$

Here, we consider a permittivity of GaAs *ε* = 12.9, an angular frequency *ω* = 2*π* · 2.3 ∙ 10^12^ Hz and the plasma frequency

2 $$\omega _P^2 = \frac{{ne^2}}{{{\it{\epsilon }}_0{\it{\epsilon }}m \ast }},$$

which contains the respective electron density *n*, the electron charge *e*, the vacuum permittivity *ε*_0_ and the effective electron mass m* = 0.067 · 9.11 · 10^−31^ kg.

### Optimization procedure

The NIR-pattern optimization is performed through a gradient-based Broyden-Fletcher-Goldfarb-Shanno (BFGS) algorithm^19,33,34^. The small fluctuations between acquisitions lead to reliable gradient estimations and enable the implementation of a gradient-based routine. Compared to algorithms used in former works^19^, here the BFGS algorithm turns out to be faster and more efficient (Supplementary Fig. 8). Experimentally, the procedure is initiated by projecting with the spatial light modulator a Hadamard basis on the device in the form of a 16 by 16 array (Step 0, inset of Fig. 2b). The corresponding spectrum is measured and the cost function estimated ($f = \frac{{I_{{\mathrm{opt}}}}}{{I_{{\mathrm{ref}}}}}$, see main text). The next NIR pattern is computed by the BFGS routine, displayed on the device and the new cost function is measured. The procedure is repeated until the cost function converges.

For a moderate voltage and a reasonable number of lasing modes (typically 9.5 V and <10 modes), the single-mode optimization performed in Fig. 2 reveals to be reproducible and robust. At higher voltage, though, the available gain and the number of modes become too important and the process—which simultaneously depletes the gain for multiple modes—loses in efficiency. Yet, the use of a stronger NIR power results in more pronounced modifications of the spectrum (Supplementary Fig. 6) and thus can be foreseen as a way to improve technical performances. Moreover, previous works^19^ have demonstrated that using larger arrays to modulate the NIR beam (i.e., array of higher dimension) would improve the performances of the optimization routine.

### Mode interactions

The mode mappings displayed in Fig. 3b and c as well as in Fig. 4b–e are performed by scanning the 270-µm spot across the device (Supplementary Fig. 2a). At each spot location, the spectrum is acquired and the intensities of the different modes are measured. For each mode, the respective map relates the changes in intensity measured for the 12 × 12 positions of the spot.

In Fig. 4, the interaction between individual mode-sensitivity maps X and Y is quantified by Pearson’s correlation coefficient, which is defined (Fig. 4a) as

3 $$\rho = \frac{{{\mathrm{Cov}}\left( {X,Y} \right)}}{{\sigma _x\sigma _y}},$$

in which Cov(*X*, *Y*) stands for the crossed covariance of *X* and *Y*, while *σ*_*x*_ and *σ*_*y*_ stand to their individual variances, respectively.

## Supplementary information

Supplementary Information

## Data Availability

The data that support the plots within this paper and other finding of this study are available from the corresponding authors on reasonable request.
