# Supplementary material for: All-optical adaptive control of quantum cascade random lasers
Source: Nat Commun. 2020 Nov 2;11:5530. doi: 10.1038/s41467-020-19305-8 (PMC7606519; doi:10.1038/s41467-020-19305-8)
Supplement: Supplementary file 1 — Supplementary Information [file 41467_2020_19305_MOESM1_ESM.pdf]

Supplementary Information: All-Optical Adaptive Control of Quantum Cascade Random Lasers

S. Schönhuber[1,2], N. Bachelard[3], B. Limbacher[1,2] M.A. Kainz[1,2], A.M. Andrews[2,4], H. Detz[5], G. Strasser[2,4], J. Darmo[1,2], S. Rotter[3] and K. Unterrainer[1,2]

1) Photonics Institute, TU Wien, 1040 Vienna, Austria

2) Center for Micro- and Nanostructures, TU Wien, 1040 Vienna, Austria

3) Institute for Theoretical Physics, TU Wien, 1040 Vienna, Austria

4) Institute for Solid-State Electronics, TU Wien, 1040 Vienna, Austria

5) Central European Institute of Technology, Brno University of Technology, 61200 Brno, Czech Republic

Supplementary Note 1: Numerical computation of the lasing modes

To evaluate the spatial extent of the lasing modes, we performed in Supplementary Fig. 1 a 3D COMSOL simulation. Panels a, b and c describe the emission of a bulk-disk device (i.e. without scattering holes). Panel a shows the refractive-index distribution in the (x, y) plane, where the blue part depicts the active region ($n = 3.6$) and the white the surroundings ($n = 1$). Panel b shows for the transverse field (E_z) an eigenstate at frequency 2.3 THz, which corresponds to a whispering gallery mode characterized by a field aligned along the disk's edge with little intensity close to the center. Panel c emphasizes that the field is confined in the (x, y) plane and its out-of-plane emission (i.e. along z) is zero.

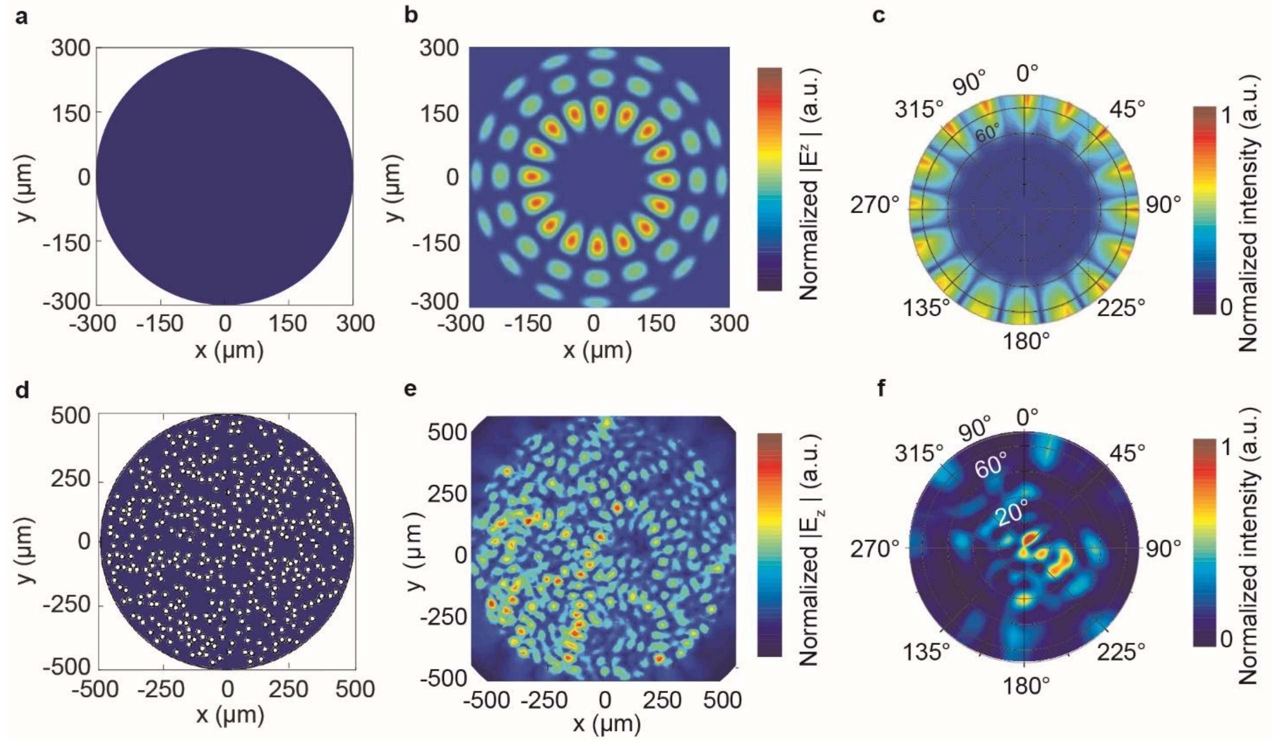

Supplementary Fig. 1 a, b and c: FEM simulation of a disk-shaped resonator. a Distribution of refractive index (blue n = 3.6 and white n = 1). **b** Field distribution of a whispering gallery mode (component E_z) at frequency 2.3 THz displaying 3 radial peaks inside the disk cavity. **c** Calculated far-field, showing emission in the in-plane direction only. **d e and f: FEM simulation of a QCRL mode. d** Distribution of refractive index (blue n = 3.6 and white n = 1), which mimics our experimental QCRL (20 µm−diameter holes, filling fraction 18 %). **e** Eigenmode of the z-component of the electric field (E_z) at a frequency of 2.3 THz. **f** Far-field angular emission of **e** with a strong contribution in the out-of-plane direction.

Panels d, e and f are computed in presence of holes. Panel d displays the refractive-index distribution of a system emulating our device. Holes of diameter 20 µm ($n = 1$, white circles) are spread in the

(x, y) plane on the active region ($n = 3.6$, blue). Panel e shows an eigenstate of E_z at frequency 2.3 THz spatially extended across the device, which emphasizes that lasing modes are not localized. Panel f displays the angular far-field emission associated with the mode in panel b. Remarkably, this mode is mainly vertically-emitting as discussed in our former work[1]. Supplementary Fig. 1 emphasizes that lasing modes are mainly governed by the random scattering provided by the holes. As shown in Supplementary Fig.1e, the spatial confinement is realized through the holes' disorder while the impact of the boundary is no longer the prevalent factor as in Supplementary Fig. 1b.

Supplementary Note 2: NIR Illumination of the QCRL

Throughout the main text, the Ti:Sapphire laser is used to illuminate the QCRL according to two distinct configurations:

- In Figs. 1, 3 and 4, the NIR beam is focused with a diameter of 270 µm and scanned across the surface (Fig. 1a). The beam scans the surface in 12x12 steps and at every position (x, y) a spectrum is recorded with the FTIR. A scheme of spots' positions across the device is shown in Supplementary Fig. 2a.
- In Fig. 2, the NIR beam is expended on a spatial light modulator to create an intensity-modulated pattern onto the device (Fig. 2a and Methods). Patterns are divided into 16x16 arrays (Supplementary Fig. 2b), in which each element corresponds to the local intensity of NIR field.

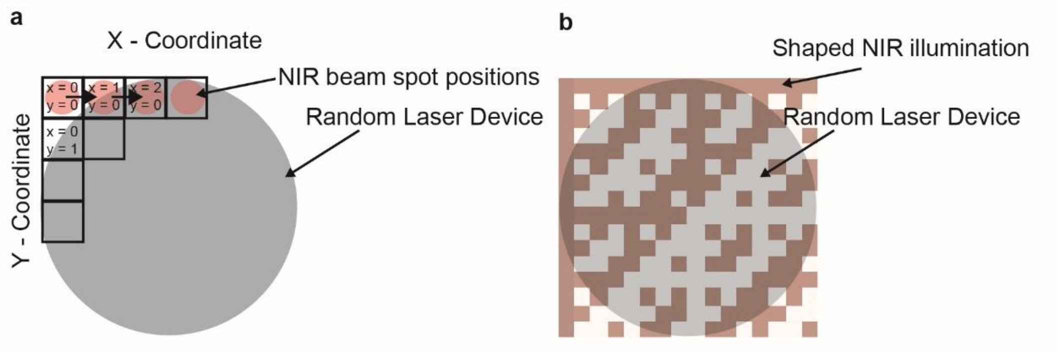

Supplementary Fig. 2 NIR illumination schemes. **a** A NIR spot with of diameter 270 µm is scanned in 12x12 steps across the QCRL device. **b** The intensity of the NIR beam is spatially modulated in a 16x16 array, in which the amplitude of each element describes the local intensity of the field.

Supplementary Note 3: Permittivity Tuning by NIR Illumination

The NIR beam locally tunes the permittivity of the active region. In Supplementary Fig. 3, a NIR spot is focused in the center of the chip (configuration of Supplementary Fig. 2a). The intensity of the Ti:Sapphire is maintained constant at 500 mW, while its wavelength is swept and the current through the QCRL is seen increasing for wavelength below the bandgap of GaAs (813 nm). When the NIR energy exceeds the GaAs bandgap, photons penetrate into the active region and produce electron-hole pairs, which results in a current increase.

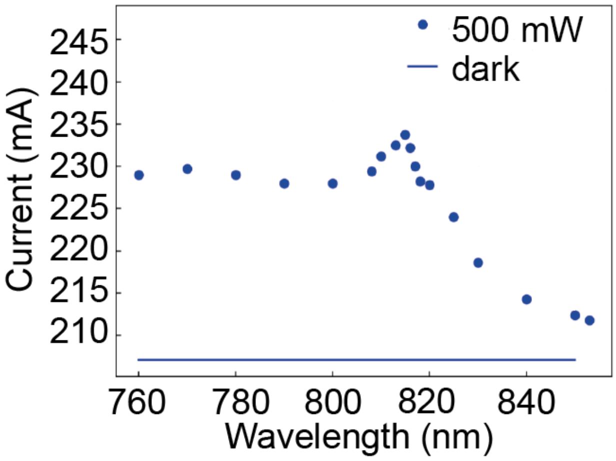

Supplementary Fig. 3 Additional current of the GaAs/$Al_{0.15}Ga_{0.85}As$ active region under constant-bias conditions and while varying NIR wavelength. The sample is cooled down to 10 K and the additional current is measured with respect to the unperturbed case. At energies below the bandgap of GaAs (wavelength $>$ 813 nm), the formation of electron-holes pairs is weak and generates a small current. At or above the bandgap (wavelength $\leq$ 813 nm), we observe a strong current increase.

Supplementary Note 4: Thermal Heating

When absorbed by the device, the NIR illumination does not produce any substantial heat increase. Supplementary Fig. 4a displays FEM simulations, in which the QCRL is modeled by a block of GaAs that absorbs the NIR light from the top. The device sits on top of a copper heat sink and is surrounded by vacuum. The Ti:Sapphire laser can emit up to 500 mW, but due to losses in the optical setup (e.g., spatial light modulator) only a fraction of this light is received by the chip. Here we assume that 100 mW are absorbed, which results in a mean temperature increase of 0.1 K. Supplementary Fig. 4b shows spectra experimentally collected while increasing device's temperature through an external thermal controller. Significant modifications in the spectra require temperature increases of more than 10 K and, thus, we deduce that the NIR-induced heating of panel a is negligible.

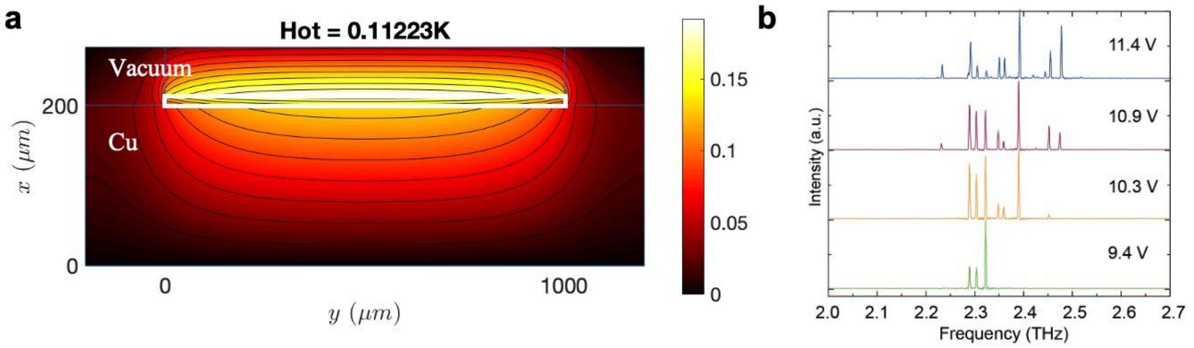

Supplementary Fig. 4 Thermal heating. a NIR-induced heating (FEM simulations). The sample is modeled by a 1000 µm-wide and 13 µm-deep rectangle of GaAs (white rectangle), which sits on top of a copper sink (Cu) while being surrounded by vacuum (Vacuum). The NIR-heating is simulated by a heat source of thickness 1 µm (corresponding to GaAs absorption length at 813 nm). The heat source mimics the absorption of an optical power of 100 mW and produces a mean temperature increase of the whole chip of roughly 0.1 K. **b** Spectra experimentally measured under an increasing bulk temperature. The QCRL is pumped under a bias voltage of 11.4 V and we observe substantial changes only for temperature increases on the order of tens of Kelvins.

Supplementary Note 5: Spectral Modifications under an Increasing Bias Voltage

Supplementary Fig. 5 displays the spectra of our QCRL device, while the bias voltage is progressively increased. We observe that raising the available gain only leads to the emergence of new lasing modes. Yet, the gain is differently shared amongst modes: the intensities of some modes lasing at low voltage (green) become proportionally smaller than the ones of modes emerging at higher voltage (e.g. blue).

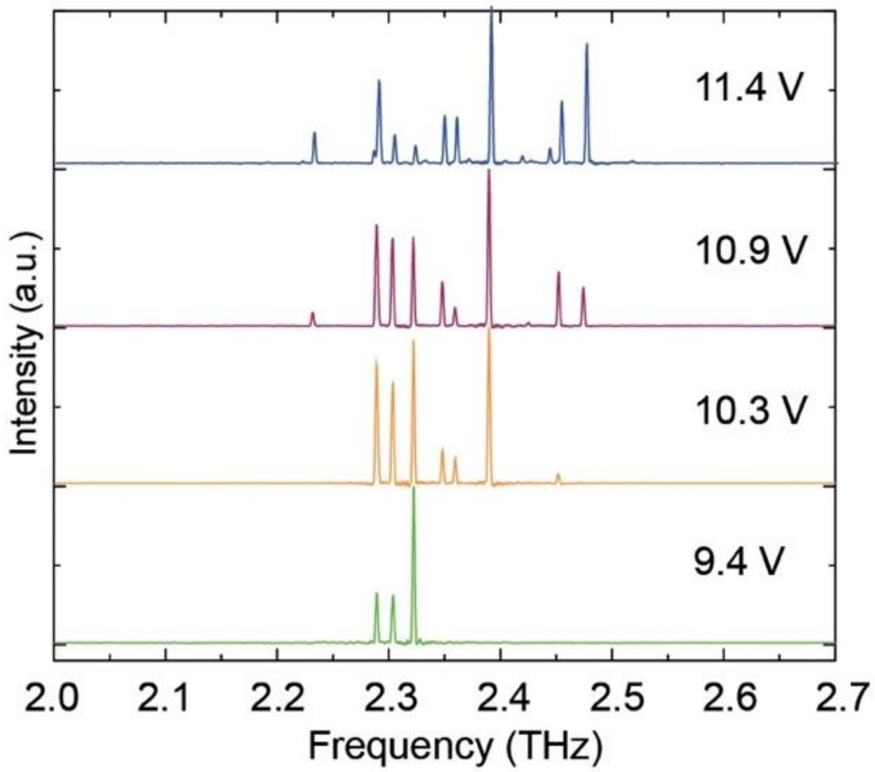

Supplementary Fig. 5 Increasing bias. QCRL spectra collected under a bias of 9.4 V (green), 10.3 V (orange), 10.9 V (purple) and 11.4 V (blue). An increase of the available gain leads to the emergence of new modes without suppressing any pre-existing ones.

Supplementary Note 6: Spectral Modifications Induced by Scanning the NIR Illumination

Supplementary Fig. 6 shows spectra measured while the system is scanned by the NIR beam (configuration of Supplementary Fig. 2a). Panels a to c show the evolution of the THz emission for a spot in the middle of the chip, while its NIR power is increased. We observe that stronger NIR power induces larger modifications. Like in Supplementary Fig. 5, the NIR beam redistributes the gain differently amongst lasing modes, which induces a modulation (i.e., increase or decrease) of intensities. The modes that are observed emerging (respectively vanishing) are brought above (respectively below) lasing threshold by the new gain distribution imposed by the NIR spot. Panel d is a color plot, in which the power is kept to 500 mW and the spot is scanned across the whole chip. It confirms that the laser emits at specific frequencies: if a mode can stop or start lasing for given positions, it always emits at the same frequency.

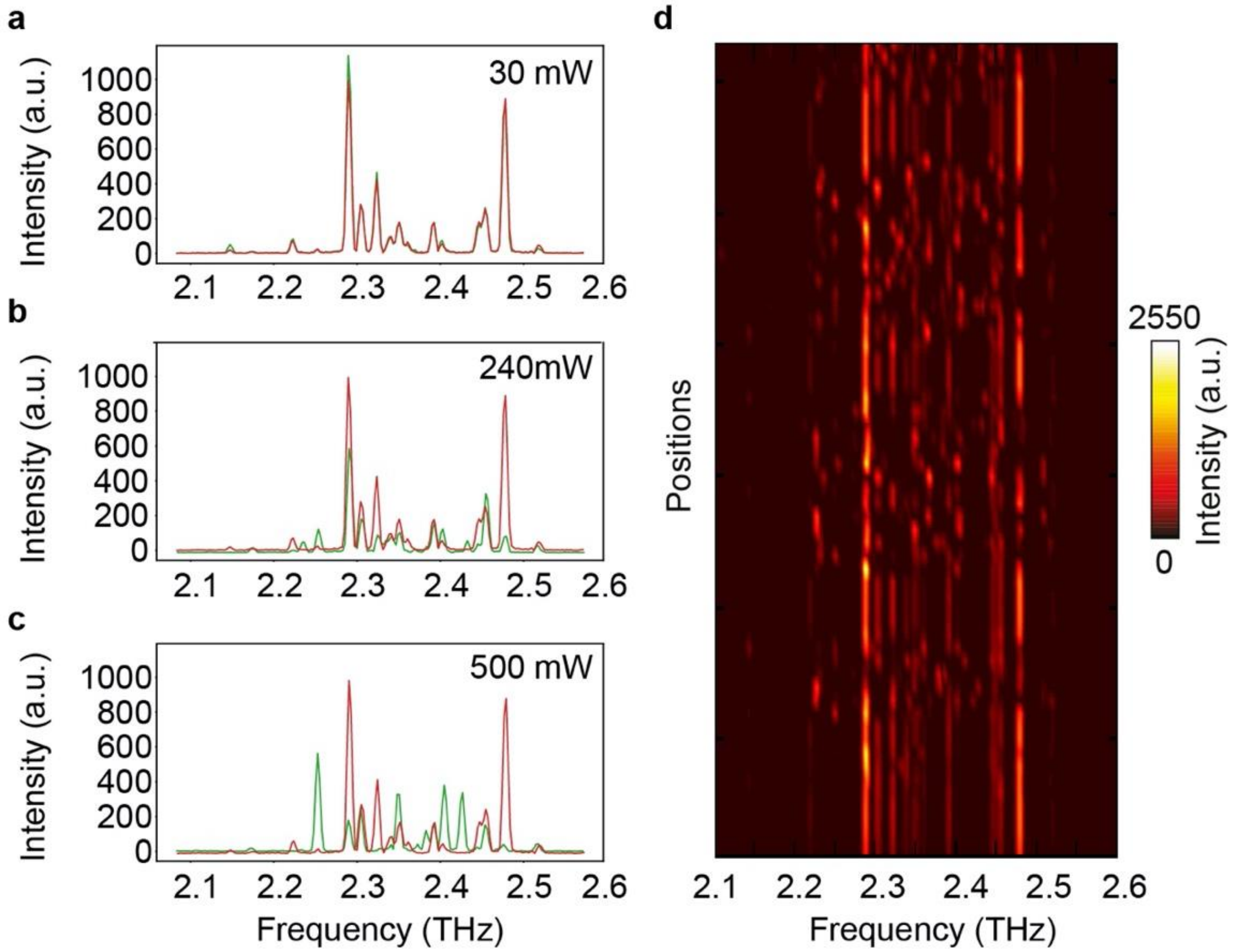

Supplementary Fig. 6 Spectral modifications under spot scanning. a Spectra of the unperturbed system (red) and under a NIR beam (30 mW) at position (5,8) (green). **b** Spectra of the unperturbed system (red) and under a NIR beam (240 mW) at position (5,8) (green). **c** Spectra of the unperturbed system (red) and under a NIR beam (500 mW) at position (5,8) (green). **d** Color plot of the spectra collected while scanning the spot across the whole sample (500 mW).

Supplementary Note 7: Integrated Lasing Intensity

Supplementary Fig. 7 shows the integrated intensity of Supplementary Fig. 6d. For each position the spectrum is summed over frequencies. The horizontal dashed line displays the intensity in absence of perturbation. The mean intensity (≈ 42000 a.u.) is decreased compared to the unperturbed case (≈ 52000 a.u.), which originates from a modification by the NIR beam of the energy levels in the gain region.

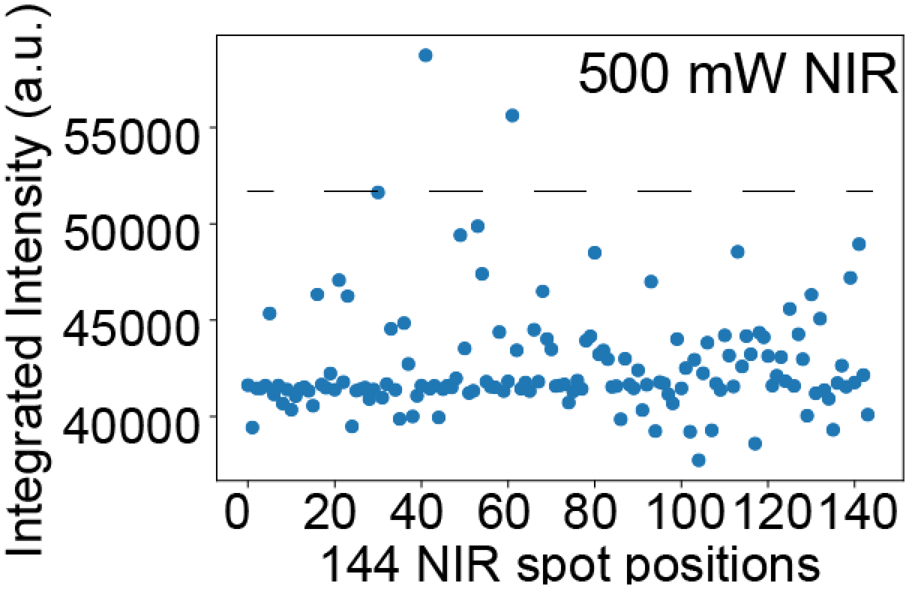

Supplementary Fig. 7 Intensity variations under spot scanning. Integrated intensity (summed over the whole spectral bandwidth) for the 12x12 = 144 NIR spot positions introduced in Supplementary Fig. 2a (500 mW). The black dashed line indicates the output intensity without NIR illumination. The mean intensity (≈ 42000 a.u.) is lower than in the unperturbed case (≈ 52000 a.u.) because of the misalignment of the energy levels in the gain region induced by the NIR field.

Supplementary Note 8: Optimization routine

In Supplementary Fig. 8, we compare two different routines for the optimization of the 2.32 THz mode displayed in Fig. 2e. Panel a shows the evolution of the cost function using a Simplex algorithm (as used in[2]), which does not rely on gradient estimations. Panel b repeats this optimization with the BFGS routine used in this work (final optimization shown in Fig. 2e). Because our approach relies on gradient estimations, it reveals both faster and more efficient.

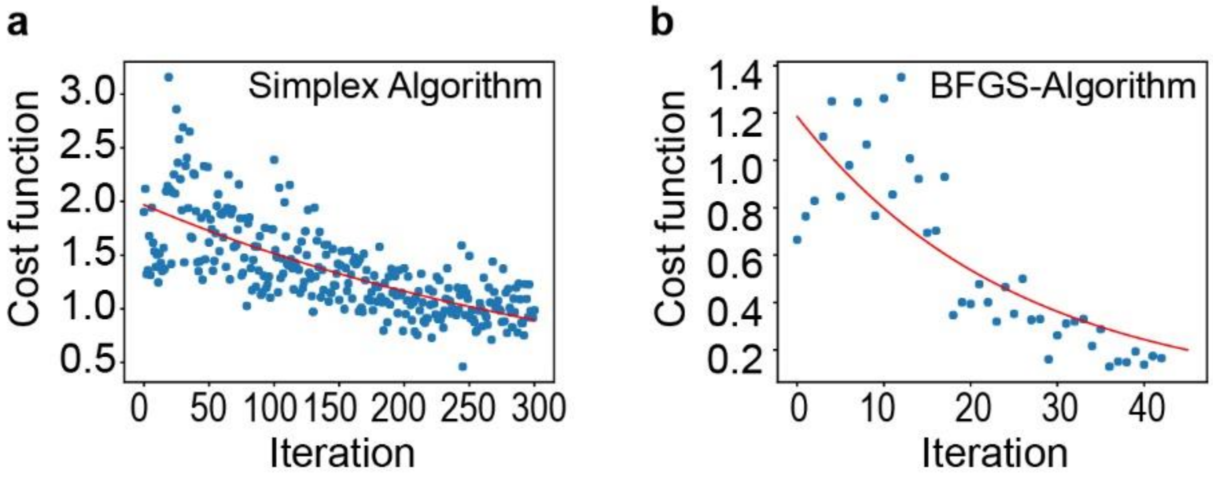

Supplementary Fig. 8 Comparison of optimization routines. Optimization of the 2.32 THz mode in Fig. 2**e** using a Simplex **a** or BFGS routine **b**. The routine used in this work **b** reveals faster and more efficient.

Supplementary Note 9: Bi-modal Optimization

Supplementary Fig. 9 shows a bi-color optimization, in which we adapted the cost function of the optimization procedure. Specifically, we note P_1, P_2 and P_3 the intensities of the three modes displayed in Panel a. We seek to select simultaneously the modes #1 and #2 while decreasing #3 and the new cost function reads $f = |P_1 - P_2| + |P_3|$. Panels b, c and d show the optimization procedure that is observed converging after 500 iterations.

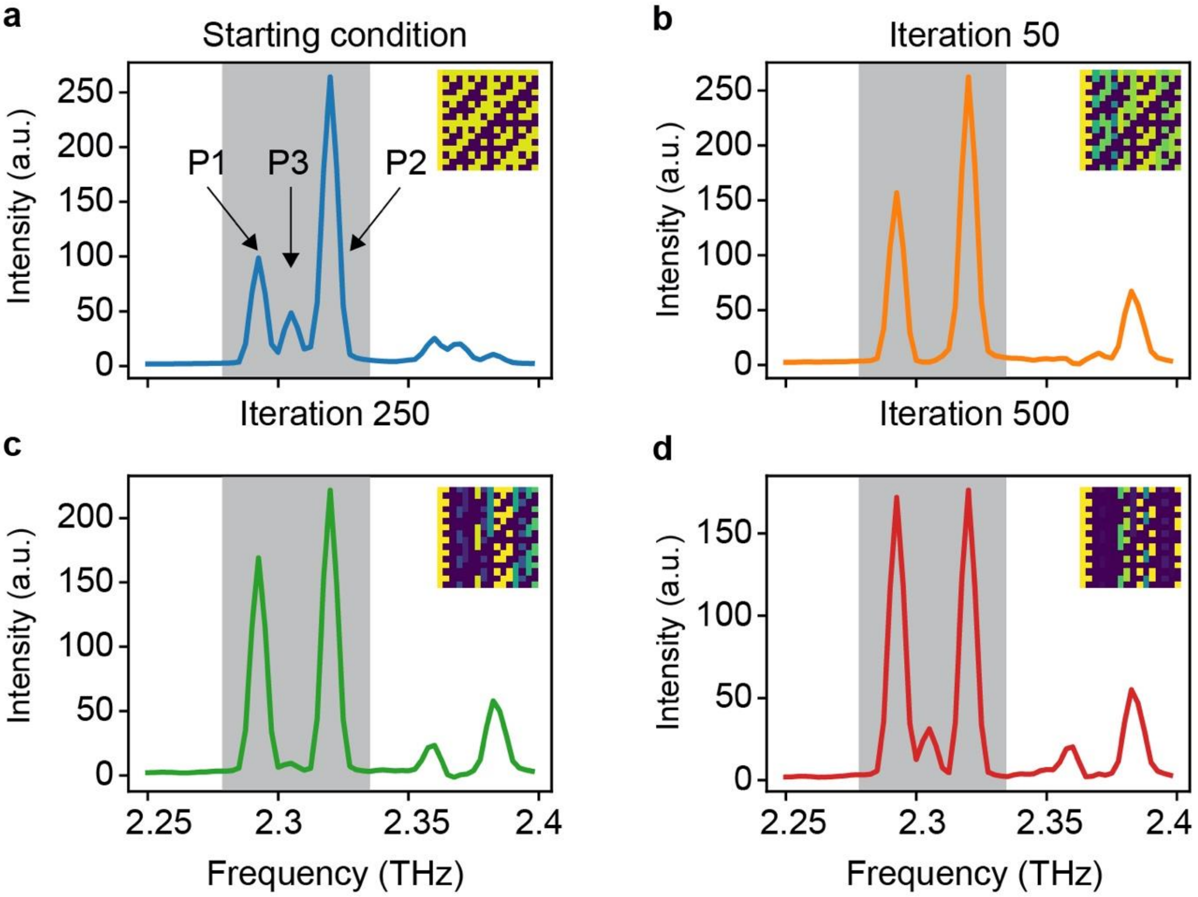

Supplementary Fig.9 Bi-modal optimization. a QCRL under a constant bias of 9.5V and illuminated by the NIR profile provided in inset. P_1, P_2 and P_3 mark the intensity of mode #1, #2, and #3, respectively. To select simultaneously modes #1 and #2, the cost function of the optimization routine aims at imposing similar intensities between modes #1 and #2 while simultaneously reducing #3, which reads $f = |P_1 - P_2| + |P_3|$. **b**, **c** and **d** Collected spectra (corresponding NIR patterns in inset) during the optimization at iteration 50, 250 and 500, respectively. By construction the cost function does not take into account others modes than #1, #2, and #3 (i.e. modes outside of the grey area).

Supplementary Note 10: Mode mapping by NIR Illumination

The mode mapping displayed in Fig. 3 and 4 of the main text are performed by scanning the NIR spot across the chip (configuration of Supplementary Fig. 2a). For instance, Supplementary Figs. 10 and 11 correspond to the mappings shown in Figs. 3b and c, respectively. For each spot position (x, y), the spectrum is collected and the intensity of individual modes are extracted. A mapping provides the intensity evolution for a mode at a given frequency (e.g. 2.29 THz in Supplementary Fig. 10) as a function of coordinates (x, y).

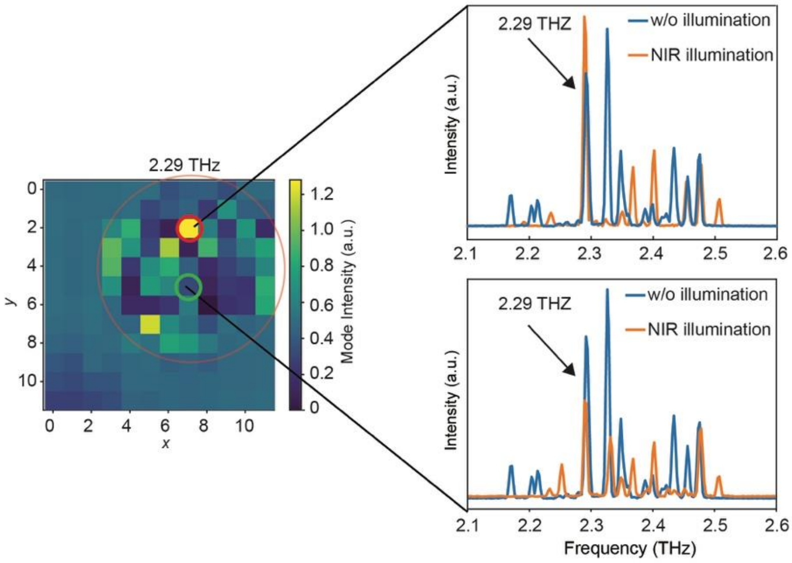

Supplementary Fig. 10 Sensitivity map of the mode at 2.29 THz (Fig. 3b). Spectra obtained for two different NIR spot positions. The rows (respectively columns) of the map correspond to the x (respectively y) positions of the NIR spot. Under the action of the NIR spot, the mode at 2.29 THz can be amplified (top spectrum) or decreased (bottom spectrum), while the gain is redistributed to other modes.

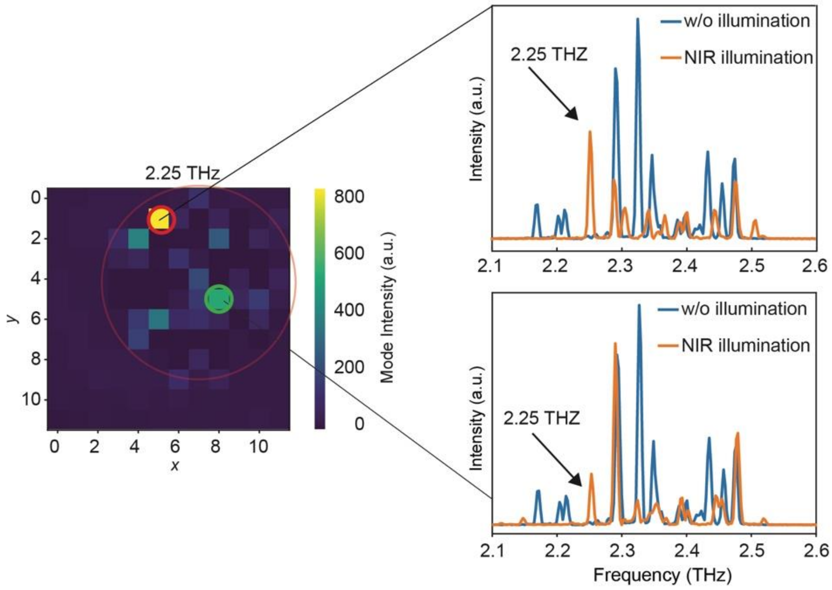

Supplementary Fig. 11 Sensitivity map of the mode at 2.25 THz (Fig. 3c). Spectra obtained for two different NIR spot positions. The rows (respectively columns) of the map correspond to the x (respectively y) positions of the NIR spot. The mode at 2.25 THz only emits for specific spot positions, see top and bottom spectra.

Supplementary References

1. Schönhuber, S. et al. Random lasers for broadband directional emission. Optica **3**, 1035 (2016).
2. Bachelard, N., Gigan, S., Noblin, X. & Sebbah, P. Adaptive pumping for spectral control of random lasers. Nat. Phys. **10**, 426–431 (2014).
